# Corn-Based Fermented Beverages: Nutritional Value, Microbial Dynamics, and Functional Potential—An Overview

**DOI:** 10.3390/foods15010027

**Published:** 2025-12-22

**Authors:** Milagros López-Reynoso, Gloria A. Martínez-Medina, Liliana Londoño-Hernández, Pedro Aguilar-Zarate, Javier Ulises Hernández-Beltrán, Ayerim Y. Hernández-Almanza

**Affiliations:** 1 Food Products Research and Development Laboratory, School of Biological Sciences, Universidad Autonoma de Coahuila, Torreón 27276, Coahuila, Mexico; milagroslopez@uadec.edu.mx; 2 School of Biological Sciences, Universidad Autonoma de Coahuila, Torreón 27276, Coahuila, Mexico; gloria-martinez@uadec.edu.mx (G.A.M.-M.); ulises.hernandez@uadec.edu.mx (J.U.H.-B.); 3 School of Basic Sciences, Technology and Engineering, Universidad Nacional Abierta y a Distancia—UNAD, Palmira 763531, Colombia; 4 Laboratorio Nacional CONAHCYT de Apoyo a la Evaluación de Productos Bióticos (LaNAEPBi), Unidad de Servicio, Tecnológico Nacional de Mexico/I.T., Ciudad Valles 79010, San Luis Potosí, Mexico; pedro.aguilar@tecvalles.mx

**Keywords:** corn varieties, nutritional value, fermented beverages, microbiological characteristics, lactic acid bacteria, bioactive compounds

## Abstract

Interest in fermented beverages has increased in recent years due to evidence showing their health benefits. In Latin America, corn is the most widely consumed cereal and stands out for its genetic diversity, cultural importance, and nutraceutical potential. This review evaluates the effect of grain pigmentation on its nutritional and bioactive composition, as well as its relationship with the production of traditional fermented beverages. Studies describing the composition of different corn varieties, fermentation processes, the microbiota involved, and safety-related aspects are considered. Evidence indicates that varieties differ in their carbohydrate, protein, mineral, and bioactive compound content, which influences the functional properties and microbiological and sensory characteristics of the resulting beverages. These beverages are produced through spontaneous or semi-controlled fermentation by lactic acid bacteria, fungi, and yeasts, which produce metabolites such as organic acids and bacteriocins that increase the nutritional and functional value and safety of the product. However, good manufacturing practices must be applied to ensure their safety. Even so, there are still gaps in our knowledge about the influence of different corn varieties on the final composition and acceptance of these beverages, highlighting the importance of further research.

## 1. Introduction

Since its origin in Mexico, corn has become one of the most widely used cereals, from pre-Hispanic times until now. It has been vital to the survival and nutrition of the population [1]. Mesoamerican cultures considered corn sacred and used it as currency for trade between settlements. Over time, this increased the popularity of corn and led to its commercialization in Europe and other parts of the world. Despite the passage of time, corn remains a symbol of identity and culture in Latin America [2,3].

Different types of corn have been developed over the years and are colloquially distinguished by their color. However, grouping by color can encompass several varieties. The term “variety” refers to a group of plants that share common characteristics, such as morphological, genetic, historical, or ecological features [4]. In the case of corn, varieties are grouped according to phenotype (shape of the cob), grain type (ability to produce popcorn), and place of harvest or relevance (Table 1). According to the Comisión Nacional para el Conocimiento y Uso de la Diversidad (CONABIO), 220 corn races have been reported in Latin America, 64 of which come from Mexico. Of those, 59 are considered native [4,5,6].

In Mexico, corn has been classified into the following seven racial complexes: Conical, Sierra de Chihuahua, Ocho Hieleras, Chapalote, Early Tropicals, Tropical Dent, and Late Maturing. These complexes are used to elaborate products such as tortillas, huaraches, tesgüino, coricos, menudo, tamales, and pozol, among others [7].

Consequently, this crop has acquired great economic importance. According to the Agri-Food Outlook for Corn, global production was projected to reach a maximum of 1228.1 million tons (MMT) by the end of 2024 and a maximum of 1220.5 MMT in 2025, with a projected consumption of 1204.1 MMT. However, only 36.8% of this consumption is intended for human and industrial use [3].

Despite the relevant nutritional, cultural, and economic importance of this crop by itself, traditional practices such as fermentation have been applied over time and across several geographical points [8]. Fermentation enhances several characteristics, such as flavor, texture, and nutritional value, but also influences safety and preservation [9]. At present, the fermented products market is growing through an increase in demand due to the strong relationship between fermented food consumption and health promotion and disease prevention, as well as the integration of traditional heritage into modern food practices [9,10]. The fermentation procedure provides an advantage over traditional food component and food production techniques, requiring reduced land, water input, and generating fewer greenhouse gas emissions compared to traditional strategies [11]. Fermented foods offer several solutions to contemporary concerns, and biotechnology could help achieve this goal during the globalization era, especially in terms of health and food security.

In Latin America, various fermented corn-based foods have been developed to incorporate this cereal into the diet in different forms and preserve it [12]. Examples include tejuino and pozol [13]. Fermented beverages are defined as those produced through the growth of microorganisms (bacteria, yeasts, and/or fungi) in a controlled or spontaneous manner. These microorganisms are responsible for degrading the substrate components through different enzymes to obtain energy [14]. Similarly, various types of corn have been traditionally used to make fermented beverages. Despite the significant cultural and social value of these products [15,16], much remains unknown about the characteristics of these beverages and how incorporating different corn varieties (with different pigmentation) affects their fermentation process and final characteristics. This review aims to analyze and synthesize information on the nutritional value and composition of different types of corn, differentiated by pigmentation, and the production of corn-based fermented beverages in Latin America. The review will also examine such beverages’ nutritional composition, physicochemical characteristics, and acceptability.

**Table 1:** Most common classifications of corn.

Morphology		References
Dent	On the periphery and rear of the grainm there is a hard, dense, translucent endosperm, while the central part is soft, white, and floury. These grains have indentations.	[17]
Flint	They have a hard, thick, and glassy endosperm, and are smooth and round grains.	[17]
Waxy	Their main characteristic is that they contain 100% amylopectin, which gives them a sticky texture and various uses.	[18]
Sweet	This type is notable mainly for its high sugar content (5–6%) and low starch content (10–11%).	[19,20]
Pop	Small, hard, round corn kernels that have the ability to accumulate pressure and pop to form popcorn.	[21,22]
Floury	It is mainly composed of soft starch with a small amount of vitreous material.	[17]
**Pigmentation**		
White	It is usually the most widely used in food, animal feed, and industry, as consumers prefer it.	[23]
Yellow/Red/Orange	Characterized by its high carotenoid content.	[24]
Blue/Purple/Black	This type of corn stands out for its high content of antioxidant compounds such as polyphenols and anthocyanins.	[25,26]
**Application**		
Food-Grade	These are ideal for dry milling and nixtamalization.	[27]
Animal feed (Silage)	Intended for animal consumption, with fewer restrictions than that intended for human consumption.	[28]
Specialty	This is produced and used for very specific purposes, usually obtained through genetic selection and crossbreeding.	[29]
**Genetics**		
Natives	Those that have been preserved by farmers through seed selection.	[30]
Hybrids	Conventional crossbreeding of seeds with different genetic material.	[31]
Transgenic	Produced through the genetic insertion of a specific gene into the corn genome.	[32]

## 2. Nutritional Composition of Different Corn Varieties Based on Their Pigmentation

In recent years, the pigmentation of different types of corn has attracted attention due to its bioactive and nutritional profiles, which represent a significant opportunity for the development of higher-quality products compared to those currently on the market.

Worldwide, the main varieties of corn used are white and yellow; however, there are little-known pigmented varieties that contain bioactive compounds such as anthocyanins, pigments responsible for those vibrant colors. Anthocyanins are water-soluble natural pigments that exhibit high antioxidant activity and the ability to scavenge free radicals [33,34]. Furthermore, antibacterial, anti-inflammatory, and anticancer effects of these pigments have been reported. The anthocyanin content in corn can vary according to the genotype, the plant’s growing conditions, or its location within the plant [35].

On the other hand, the presence of phenolic compounds has been reported in cereals, including corn, which is an important source of ferulic acid, a phenolic acid derived from cinnamic acid. Ferulic acid exhibits antioxidant activity by inhibiting the formation of reactive oxygen and nitrogen species, as well as neutralizing free radicals. Likewise, this compound presents antimicrobial and anti-inflammatory activities. In corn kernels, ferulic acid can be found bound to arabinose residues derived from arabinoxylans, which may enhance the antioxidant capacity of intestinal epithelial cells due to the production of short-chain fatty acids and the release of ferulic acids during the fermentation process [36,37].

Corn is the cereal with the highest carotenoid content, with zeaxanthin and lutein present at the greatest concentrations. These pigments are located primarily in the endosperm of the grain, giving it yellow, red, or orange colors [38]. The main beneficial effect associated with carotenoids is their defense against oxidative stress and their ability to scavenge free radicals [37,39]. Likewise, the carbohydrate, protein, ash, and lipid content may vary depending on the corn pigmentation.

### 2.1. Carbohydrates

Consumption of this cereal is important in terms of energy because carbohydrates are the primary source of energy for humans. Corn (*Zea mays* L.) contains about 70% carbohydrates (CHO), which is stored in the grain’s endosperm [40]. White corn (*Zea mays* L.) is one of the most widely grown varieties in Mexico [41]. According to Abiose & Ikujenlola [42], white corn has a carbohydrate content of 73%, like the content reported by Rouf Shah et al. [43]. However, it is important to note that grain composition depends on factors such as growing conditions, harvest, irrigation water, climate, and genetics, etc. [44].

For example, according to a study conducted by Majamanda et al. [45] which analyzed orange, red, and purple corn varieties, it was found that the purple variety had a higher carbohydrate content, at 65.52%, while the red variety had 58.73%. Libron et al. [46], on the other hand, reported maximum values of 77.75% and 71.30% for orange varieties APN-1042 and APN-426, respectively. The other varieties (yellow, red, and purple) fall within this range. These findings align with those reported by Mex-Álvarez et al. [47], ranging from 75.07% to 70.7%. Considering that pigmented varieties tend to have higher carbohydrate content, the range of CHO content is very broad and diverse. This makes it difficult to define specifically based only on grain pigmentation. Therefore, other factors such as growing region, harvest, and specific variety and breed should be considered.

#### 2.1.1. Starch

Starch, a type of carbohydrate composed of amylose and amylopectin, is the main source of energy for plants. It is stored in granules and is the primary component of corn kernels [48,49]. There are two types of starch: resistant and digestible. Digestible starch is hydrolyzed and digested by the intestine, while resistant starch reaches the colon intact [50]. Blue and black corn varieties have been reported to have higher starch content than white corn [48,51]. Agama-Acevedo et al. [52] reported that black corn has a higher percentage of damaged starch (DS) (10.8%) than white (6.4%) and blue (3.9%) corn. This can affect the final characteristics of the end product and the usable starch fraction, regardless of total starch content. For instance, Lakshmi et al. [53] found that white corn has a higher total starch content (TS) but contains a higher proportion of damaged starch. Meanwhile, orange and purple corn contain 52–54% total starch, less than 3.5% damaged starch, and a higher resistant starch content (RS) compared to all other types of grain analyzed. Conversely, Rojas & Pacheco [54] found that yellow corn has a higher TS content and lower RS content than purple corn. Therefore, when using corn for food production, it is important to consider the quality of the starch present in the grain, how it is preserved during processing, and its resistant starch content. Resistant starch has been shown not to raise the glycemic index of consumers and to preserve the grain’s technological characteristics [55].

#### 2.1.2. Fiber

The Food and Agriculture Organization (FAO) defines dietary fiber as all components of a plant-based diet that cannot be hydrolyzed in the intestine. It is also defined as the sum of polysaccharides that are neither starch nor lignin [56]. There are two types of dietary fiber: soluble and insoluble, depending on their ability to dissolve in water [57]. Corn primarily contains arabinoxylans, heteroxylans, phenolic acids, and cellulose, which are typically found in the pericarp of the grain [58]. In general, pigmented corn varieties tend to have higher fiber content. Rodríguez-Salinas et al. [59] found that yellow corn has a fiber content of 1.76%, followed by white corn (1.69%), purple corn (1.63%), and red corn (1.41%). This coincides with the findings of Çetin-Babaoğlu et al. [60], who also report high fiber content in blue corn, followed by yellow and red corn. Camelo-Méndez et al. [61] also report a high fiber content for blue corn. Generally, the fiber content of blue and purple corn is statistically equal [62]. Blue and purple corn tend to have higher fiber content than other varieties because the pericarp of these grains contains higher amounts of non-starch polysaccharides [63,64].

### 2.2. Proteins

Proteins are biomolecules composed of amino acids. They are the second most abundant component in corn, with zein being the main protein, accounting for 50% of the total proteins in this cereal [65]. Most varieties have a similar total protein content within the range of 10.95–13.12% [45,66]. Similarly, Mansilla et al. [67] found similar results between white and purple varieties. However, white varieties tend to have higher protein and lipid content. Özdemir et al. [68] found that the protein content was highest in non-pigmented (white) corn, followed by purple, yellow, and red. Thakur et al. [69] also found different protein content levels in yellow, white, and red varieties, with red varieties having the highest content (7.76%) and yellow varieties having the lowest (6.29%). Specifically, this study highlights a positive correlation between protein content and the “a” value in the L*a*b color system.

Not only is it important to highlight the total protein content, but the quality of the proteins present in the grain, in other words, its amino acid profile, should also be emphasized [70]. Blue corn has a higher tryptophan content, which makes its protein superior compared to red corn. Additionally, environmental conditions and crop management have been shown to directly impact the nutritional characteristics of corn [70]. Currently, there are high-quality protein varieties, or Quality Protein Corn (QPC), developed to increase essential amino acid content, such as tryptophan and lysine [71].

### 2.3. Fats

Corn is one of the cereals with the highest lipid content, ranging from 3 to 5%. However, corn is not considered an oilseed because approximately 80% of its fats are retained in the germ of the grain. To be considered an oilseed, a grain should contain over 7% fat [40,72]. The fat content of different corn varieties is primarily influenced by genetic and climatic factors related to grain pigmentation, particularly genetic expression [53]. The fat composition of different pigmented corn varieties has been reported to range from 3.38 to 6.15 g/100 g [59]. For instance, Agama-Acevedo et al. [51] examined the proximate composition of white, blue, and black corn and found that white corn had a higher fat content (4.8%) than blue and black corn, which were statistically equivalent. Similarly, Lakshmi et al. [53] studied seeds of different colors, finding values of 5.9% for purple corn and 4.12% for white corn, the lowest value. These results are consistent with those of Peña-Betancourt et al. [73], who studied the proximal composition of various corn varieties from Mexico. They analyzed blue, white, yellow, and red varieties, finding a lipid composition range of 4.4–5.66 g/100 g of sample. This variability is a consequence of the corn’s genotype and processing [40]. It is important to highlight the importance not only of total lipid content, but also of fatty acid composition (quality) [73].

The World Health Organization (WHO) recommends that total energy intake from fats be 30% or less, with less than 10% coming from saturated fatty acids [74]. The WHO also recommends consuming unsaturated fats instead of saturated and trans fats [75].

Corn is composed mainly of unsaturated fatty acids. As shown in Table 2, the fatty acid composition varies among different pigmented corn varieties. The fatty acids found in the highest proportions in this cereal are linoleic and oleic acids [72]. Linoleic acid is an essential fatty acid because the body cannot synthesize it metabolically; therefore, it must be obtained through diet [76]. Linoleic acid has been proven essential for preventing skin lesions, growth retardation, and thrombocytopenia [77]. However, excessive consumption can lead to health problems, so consumption of 1–2% of energy is recommended [78]. Oleic acid, on the other hand, is a monounsaturated fatty acid that reduces inflammation caused by other fatty acids, insulin resistance, and cholesterol (low-density lipoprotein) levels. It also reduces the risk of cardiovascular disease [79,80,81].

### 2.4. Ash (Minerals)

Although they are classified as micronutrients, minerals are essential to human nutrition and health. They maintain osmotic pressure within cells, regulate enzyme activity, participate in the structure of the human body, and perform other functions [86]. Therefore, the quantity and quality of sources from which minerals are obtained are of the utmost importance for maintaining good metabolic balance and function. Corn contains approximately 1.3% of ash, 78% of which is found in the germ. This cereal is mainly composed of phosphorus (P), potassium (K), magnesium (Mg), zinc (Zn), iron (Fe), copper (Cu), and sodium (Na) [86].

Total ash content has been reported to be around 150 mg/100 g for yellow varieties, 107 mg/100 g for blue varieties, 138 mg/100 g for red varieties, 136 mg/100 g for purple varieties, and 118 mg/100 g for white varieties [46,87,88]. Similarly, the highest concentrations of elemental components such as magnesium, sodium, and sulfur have been reported in purple genotypes [88]. The calcium content ranges from 24.58 to 29.17 mg/100 g in purple varieties and from 28.49 to 31.62 mg/100 g in white, yellow, and red varieties [59]. The potassium content ranges from 325.72 to 397.18 mg/100 g in purple varieties and from 314.20 to 338.65 mg/100 g in white, yellow, and red varieties. Iron content ranges from 2.23 mg/100 g in purple varieties to 2.50 mg/100 g in yellow varieties and 1.72 mg/100 g in white varieties [87,88]. This variability is mainly due to the genotype of each corn variety and its impact on chemical composition.

## 3. Bioactive Compounds in Different Corn Varieties

These varieties contain various bioactive compounds, including phenolic compounds, anthocyanins, and carotenoids. These compounds have been shown to have nutraceutical properties, including antioxidant, anti-inflammatory, and anti-stress effects, as well as improvements in cardiovascular and gastrointestinal health [37].

### 3.1. Phenolic Compounds and Anthocyanins

As some of the most abundant components in plants, including cereals, fruits, and vegetables, phenolic compounds are considered secondary metabolites. In recent years, the importance of phenolic compounds has been recognized and demonstrated, not only for their impact on the organoleptic characteristics of numerous foods, such as color and smell, but also for their health benefits as antioxidants and regulators of cellular activity [89].

Cereals usually contain phenolic acids, such as ferulic, coumaric, and gallic acids, as well as flavonoids, mainly anthocyanins. Anthocyanins are pigments that produce blue-purple colors [90]. These components are found in free form or bound to the cell wall [91]. Corn is one of the cereals with a high percentage of these compounds, and it is more abundant in pigmented varieties [37].

The composition of each corn variety is highly variable, as is the composition within groups of corn with similar pigmentation (Table 3). A study by Özdemir et al. [68] characterized white, yellow, red, and purple corn varieties and found that pigmentation plays a significant role in the composition of bioactive compounds. This coloration can serve as an indicator of the types of compounds present in the raw material. Similarly, Rodríguez-Salinas et al. [59] demonstrated a positive correlation between total polyphenol content and anthocyanin content. On the other hand, Zilic et al. [92] emphasized the importance of considering the composition of these varieties when selecting the most suitable raw material for developing new products with desired, or even improved, characteristics.

### 3.2. Carotenoids

Carotenoids are a different type of pigment than anthocyanins. They give foods a yellow or red color and must be obtained through diet. Foods that contain carotenoids include carrots, tomatoes, squash, papaya, and yellow corn, among others. The most common carotenoids in food are β-carotene, lutein, zeaxanthin, and lycopene [101]. The hardness of yellow and orange corn kernels, in terms of protein content, has been reported to be directly related to the corn’s ability to store certain carotenoids [102]. In this study, the “Flint” variety was found to contain more lutein, α-cryptoxanthin, and α-carotene than the dent variety [102].

Additionally, kernel color directly influences carotenoid content, which depends on corn genotype [103]. Similarly, corn variety, breed, time of harvest, and ripeness can increase or decrease carotenoid content [104]. Promoting the consumption of these pigmented corn varieties (yellow corn) and incorporating them into the formulation and production of other foods increases their nutritional value and organoleptic characteristics [105].

As shown in Table 3, carotenoid content is typically only reported for yellow varieties because white or non-yellow corn usually contains minimal amounts. Yellow corn is also considered the main source of carotenoids such as lutein, zeaxanthin, and α-cryptoxanthin [94].

## 4. Fermented Beverage Production in Latin America Based on Different Corn Varieties

### 4.1. Pozol

**Figure 1:**
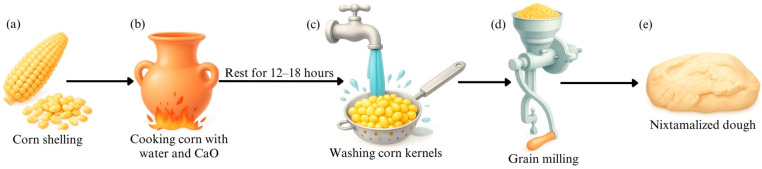
Nixtamalization process consists of the following steps: (**a**) shelling the corn; (**b**) cooking the corn with calcium oxide and leaving it to resto for 12–18 h; (**c**) washing the nixtamal to remove excess lime; (**d**) grinding the nixtamal; and (**e**) obtaining the nixtamalized dough.

Pozol, also known as “pochotl” or “pozolli” in Nahuatl, is a drink originating from Tabasco, Mexico. It emerged during pre-Hispanic times within the Mayan Empire. Due to its nutritional value, pozol is considered suitable for consumption by a wide range of people [106]. To make this drink, corn kernels are nixtamalized (Figure 1) with lemon, ground into a dough, shaped into a sphere, wrapped in banana leaves, and fermented at room temperature for 1–4 days. Finally, the fermented dough is suspended in water [107,108,109,110]. In a study conducted by Reyes-Escobar [111] in which different treatments for blue and red corn flour and different pozol preparation formulations were tested, it was found that the protein, fat, ash, moisture, and carbohydrate contents of blue corn pozol were 3.26–3.38%, 6.43–7.98%, 0.09–0.23%, 4.45–7.53%, and 80.88–85. For red corn, the respective values were 3.53–4.19%, 7.14–9.64%, 0.23–0.27%, 5.14–7.34%, and 68.48–75.98%. The variability in the final composition was mainly due to the genotype and composition of the corn and the treatment to which the corn flour was subjected.

Similarly, total phenol and flavonoid content is higher in pozol made with blue and purple corn, with reported values of 625.54–626. 37 mg FAE/100 g and 23.66–25.07 mg QE/100 g, respectively, compared to red corn pozol (584.40–603.97 mg FAE/100 g and 21.16–24.16 mg QE/100 g). Nixtamalization also increases the nutritional value of blue and red corn, and incorporating pigmented varieties increases the beverage’s content of phenolic compounds and flavonoids [111].

As an indicator of the fermentation process, the pH of pozol should decrease, while the titratable acidity should increase. The pH of pozol is around 4 and the titratable acidity, the percentage of lactic acid, is between 0.3 and 0.45% [112]. The drink is considered acceptable, as panelists tend to describe it as “I like it,” with little difference in results between varieties and procedures [111,113]. It is important to note that the pH and titratable acidity of the drink reach limits that are acceptable to consumers (pH < 4). Likewise, Jiménez-Vera et al. [114] reported that white pozol (fresh and fermented) is preferred more than pozol with added cocoa and coconut. However, the drink is mostly made with white corn because it is perceived as more appealing to consumers, giving the drink a milder flavor than that made with yellow or blue corn [115].

### 4.2. Atole Agrio

Atole agrio is a drink mainly consumed in the Mexican states of Tabasco, Chiapas, and southern Veracruz. It can be made with either white or yellow corn. According to Sánchez Cortés et al. [116] and Väkeväinen et al. [117], the typical preparation process involves soaking the kernels in water for one to five days, depending on the desired level of acidity. Then, the mixture is strained with a cloth, the pulp is reserved, and the resulting liquid is cooked. Once the liquid boils, the reserved pulp is added, followed by cinnamon, panela, and cloves. It is important to note that the preparation process may vary by region. Atole agrio contains 8.14% moisture, 10.84 g/100 g of protein, 2.41 g/100 g of fat, 78.25 g/100 g of carbohydrates, and 2.36 g/100 g of ash [118]. Atole agrio has been shown to have high antioxidant capacity, which benefits health by neutralizing free radicals [119].

Using pigmented corn varieties to prepare atole increases its nutritional value [120]; specifically, they increase the content of anthocyanins and phenolic compounds in the final product. However, the production process reduces this composition, and a high content of anthocyanins and phenolic compounds does not necessarily imply a high antioxidant capacity, because these components can be damaged during processing. Therefore, preserving the quality of the phenols and anthocyanins present is vitally important [121].

### 4.3. Tejuino

The word tejuino comes from the Nahuatl word “tecuintl”, meaning “heartbeat” [122]. Tejuino is a drink produced in Mexican states such as Jalisco, Chihuahua, Nayarit, and Sonora. Tejuino is commonly prepared using two different methods [123,124]. The first method involves germination. First, the corn kernels are germinated in the dark until the root appears. Then, they are dried in the sun for two to three days. Next, they are ground and mixed with water. The mixture is left to settle, and the liquid is separated from the sediment. The liquid is boiled with piloncillo, while the sediment is left to ferment for 24 h at room temperature. Then, the two parts are mixed and filtered. The second production process involves using nixtamalized dough, mixing it with water, and blending it. The mixture is placed over medium heat, piloncillo is added, and it is left to homogenize. Then, the juice of one lemon is added, and the mixture is boiled for an additional 20 to 45 min. This mixture is known as tejuino dough. Finally, the dough is left to rest for two days, after which it is dissolved in water at a ratio of 20% of the total volume [106,125]. Some studies report using starters; in this case, “old” tejuino is used, which has been previously prepared [126,127].

For this beverage, reported values include a pH of 2–5, titratable acidity of 0.4–1.22 meq LA (lactic acid)/L, and an alcohol content of 2–20%. However, it tends to be less than 5% in most cases [128]. In terms of its chemical composition, the following values have been reported: moisture (83–93%), carbohydrates (49–80%), protein (1.2–19%), fat (2–9.5%), and ash (0.5–4%). These differences in composition result from the tejuino production process, in other words, whether nixtamalization or germination is used [129].

### 4.4. Beers

#### 4.4.1. Chicha

Chicha is defined as a fermented, corn-based beverage. It originated in pre-Columbian America within the Inca Empire. It is currently consumed in Ecuador, Colombia, Peru, and Bolivia. The process involves soaking the corn for 24 to 48 h, allowing it to germinate for three to five days, drying it, grinding it, and fermenting it for two to three days to produce a mild beverage with low alcohol content. For a stronger beverage with a higher alcohol content, fermentation is extended to seven days. The beverage is then boiled for the same period. The beverage is then sweetened, spices are added, and it is filtered if desired, resulting in chicha [130]. According to Pezo-Torres [131], chicha has an average protein content of 0.43%, moisture content of 92.95%, crude fiber content of 0.16%, fat content of 0.19%, ash content of 0.17%, and carbohydrate content of 6.07%. Although chicha is usually prepared with white, yellow, and purple corn, very few studies have reported its nutritional profile.

The most notable physicochemical characteristics of corn chicha, particularly the purple variety (*Zea mays* L.), are a pH level ranging from 3.2 to 5.08, a Brix level ranging from 1.63 to 3.43, a titratable acidity level ranging from 0.23 to 0.36 g of lactic acid per 100 milliliters, and an alcohol percentage ranging from 2.5 to 11.70%. The composition of corn and its flour, as well as the production process, must be considered in relation to nutrient retention and, consequently, nutritional quality [132,133]. Variability in physicochemical characteristics may result from the characteristics of the raw material, fermentation time, composition of the starter, and addition of sugar during and after fermentation. Thus, standardizing manufacturing processes for this beverage is essential. Piló et al. [134] studied the final characteristics of this beverage made with white, yellow, and purple corn. Their results showed pH values below 4.5 and glycerol values of 3.65 g/L. These values can negatively impact the beverage’s sensory quality, because the high presence of organic acids and glycerol produces an unpleasant, rancid taste.

#### 4.4.2. Sendecho

Sendecho, also known as Sende, is a traditional drink of pre-Hispanic origin from the Mazahua ethnic group in Mexico. The production process involves soaking and germinating corn, drying it at 60 °C for four hours, toasting it for five minutes, and separating the sprouts from the kernels. The grains are then crushed and ground. To make the drink, 50 g of corn is added to one liter of water and boiled for two hours. Finally, 0.03% inoculum is added. The mixture is then filtered, bottled, and stored [135,136,137].

Romero-Medina [138] reported similarity between the volatile compounds present in barley and corn malt, red and blue, which makes this substrate a viable alternative for beer production. However, compounds within the phenol and terpene groups allow for differentiation between them. According to Hernández-Domínguez et al. [136], consumers prefer the drink made with blue corn, cinnamon, and pulque because it is described as more pleasant and smooth. In contrast, drinks made with yeast are perceived as having stronger smells and flavors. Similarly, introducing this type of product to consumers is complicated, as they tend to dislike changing from a product they already know or trying a new one. Nevertheless, consumers have responded positively to this product when corn of different colors is added as an adjunct [139,140]. Currently, there is no information on the basic nutritional composition of Sendecho.

## 5. Fermentation Effects in Corn and Development of Microorganisms

### 5.1. Microorganisms in Fermented Beverages Based on Different Varieties of Corn

Fermented corn-based beverages are traditional products of great cultural, nutritional, and biotechnological importance in various regions of Latin America. Examples of these beverages include pozol, tejuino, chicha, atole agrio, and sendecho. They are made from different varieties of corn (*Zea mays*) through spontaneous or semi-controlled fermentation processes. Preparation methods vary by region and community and may include steps such as nixtamalization, cooking, grinding, and mixing with water before fermentation under ambient conditions. These traditional practices result in beverages that vary greatly in flavor, texture, acidity, and functional content. The type of corn used (e.g., white, purple, or yellow) and cultural fermentation practices directly influence the microbiological composition of the final product [8,106,141]. The microbial diversity present in fermented beverages defines not only their texture, flavor, and odor characteristics, but also their stability, safety, and nutritional value.

Corn fermentation creates a complex microbial environment in which lactic acid bacteria (LAB), yeasts, filamentous fungi, and, occasionally, contaminating or pathogenic microorganisms interact. These microorganisms colonize the fermentation substrate from the grain itself, the utensils used, and the environment in which fermentation takes place. LAB such as *Lactiplantibacillus plantarum*, *Leuconostoc mesenteroides*, and *Weissella confusa* predominate in most beverages and are responsible for acidifying the medium. This inhibits the growth of unwanted bacteria and prolongs the product’s shelf life [142,143].

Yeasts such as *Saccharomyces cerevisiae*, *Candida* spp., and *Pichia* spp. participate in the production of aromatic compounds and alcoholic fermentation in beverages like tejuino and chicha. These microbial groups interact synergistically to transform corn starch into simple sugars, organic acids, vitamins, and other metabolites with functional properties [144,145]. Table 4 summarizes the main microbial communities in corn fermented beverages.

In recent years, one of the most studied aspects of these fermented beverages has been their ability to generate bioactive compounds that have beneficial effects on human health. During fermentation, lactic acid bacteria (LAB) and yeasts transform complex carbohydrates in corn into more digestible products and produce bioactive compounds. They also release peptides with antioxidant activity, synthesize B vitamins, and produce exopolysaccharides that may have prebiotic effects [35,160]. Similarly, the enzymatic activity of these microbial consortia modulates the beverages’ sensory profile, generating attributes such as acidity, effervescence, residual sweetness, and fruity or lactic aromas. This sensory transformation is essential for the cultural acceptance and regular consumption of these beverages.

#### 5.1.1. Lactic Acid Bacteria (LAB)

Lactic acid bacteria (LAB) are among the most important microbial groups involved in the fermentation of traditional corn-based beverages. Their metabolic activity is essential for substrate transformation, product stability, sensory development, and food safety. These bacteria participate in spontaneous fermentation and are found naturally in corn kernels, the environment, and traditional utensils used to make fermented beverages, such as pozol, sour atole, and chicha [106,142].

During fermentation, LAB metabolize available carbohydrates in nixtamalized or cooked corn dough, primarily glucose. This process converts the carbohydrates into organic acids, reducing the medium’s pH, inhibiting the growth of undesirable microorganisms, and providing stability and sensory properties to the product [161]. They also produce compounds such as exopolysaccharides (EPSs), bacteriocins, and B vitamins, which serve technological, nutritional, and bioactive functions [106,143,160,162]. Strains of *Lactobacillus plantarum*, *L. fermentum*, *Pediococcus pentosaceus*, and *Lactococcus lactis* have demonstrated potent inhibitory activity against *Listeria monocytogenes*, *Escherichia coli*, and *Staphylococcus aureus* through the production of bacteriocins and organic acids [153,156,163].

Recent studies have identified several species of the genus *Lactobacillus* (now classified into different genera) as predominant in beverages such as pozol, tejuino, champús, chicha de jora, and atole agrio, including *Lactiplantibacillus plantarum*, *Limosilactobacillus fermentum*, *Lactobacillus delbrueckii*, and *Lactobacillus casei* [117,142,159,164,165,166]. Similarly, heterofermentative genera, such as *Leuconostoc* and *Weissella*, have been identified in the initial stages of fermentation. These genera generate CO_2_ and aromatic compounds, which enrich the sensory profile of beverages, such as champús, Ecuadorian chicha, and atole agrio [117,143,155]. *Pediococcus* and *Enterococcus* species have also been reported less frequently and have the potential to be used as starter cultures and functional agents [117,167].

The diversity of LAB is influenced by the type of corn (e.g., creole, purple, white, or yellow), the degree of grinding, nixtamalization, and environmental conditions during the fermentation process, including temperature and time. The use of artisanal clay or wooden containers, which act as microbial reservoirs, also influences LAB diversity [168,169]. For instance, a high structural biodiversity of LAB has been reported in pozol, a traditional fermented product from southeastern Mexico. This is attributed to the high initial pH due to nixtamalization and oxygenated conditions that allow for microbial coexistence [164]. Colombian champús are dominated by a bacterial community of *Lactobacillus*, *Weissella*, and *Leuconostoc*, which is related to active lactic fermentation and the beverage’s characteristic organoleptic profile [154].

Given the advantages of using starter cultures in both the process and the product, it has been suggested that native lactic acid bacteria (LAB) be used to develop probiotic and symbiotic fermented foods that preserve the cultural identity of these traditional beverages [144]. Their taxonomic, functional, and genetic characterization has revealed their biotechnological potential, creating opportunities to enhance the safety, standardization, and added value of these ancestral products.

#### 5.1.2. Yeast and Fungi

Classified as safe, yeasts and fungi play a crucial role in the microbial and functional dynamics of fermented corn-based beverages. They participate in traditional fermentation processes by producing ethanol, carbon dioxide (CO_2_), volatile compounds, and hydrolytic enzymes that degrade starch and other macromolecules present in corn. These microorganisms transform available substrates into fermentative compounds and contribute to the development of characteristic aromas, flavors, and textures that determine the sensory acceptance of the final product [144,155].

In beverages such as champús, chicha, and pozol, the presence of yeasts such as Saccharomyces cerevisiae and other yeasts such as *Pichia fermentans*, *Pichia kudriavzevii*, *Candida tropicalis*, *Hanseniaspora opuntiae*, *Torulaspora delbrueckii*, and *Wickerhamomyces anomalus* has been documented [143,155,170]. These species can produce volatile compounds that impart fruity and floral notes, as well as exhibit amylolytic and proteolytic activity, which facilitates the release of fermentable sugars from corn [161]. The *S. cerevisiae* species is particularly predominant in chicha de jora. In addition to its fermentative efficiency, this species has been observed to be genetically adapted to hostile environments, showing tolerance to ethanol, acidity, and low nitrogen levels [134,144].

The natural fermentation of these beverages is usually dominated by mixed consortia of lactic acid bacteria (LAB) and yeasts, whose interactions enhance the microbial stability and organoleptic quality of the product. For instance, combinations of *Lactiplantibacillus plantarum* with *S. cerevisiae* or *Pichia* spp. increase the production of aromatic esters, organic acids, and bioactive compounds, thereby improving the sensory profile of beverages such as tejuino and champús [145,160]. Similarly, yeast strains isolated from traditional fermented beverages have demonstrated antimicrobial activity against *E. coli*, *Salmonella*, and *Staphylococcus aureus*. This activity is attributed to the production of ethanol, organic acids, and volatile compounds [106].

Filamentous fungi have been detected less frequently, yet are still relevant under certain conditions. Active fungal proteins related to carbohydrate and energy metabolism were identified in pozol, mainly from the genus *Aspergillus*, indicating metabolic participation in prolonged fermentations [145]. Conversely, some studies report antifungal activity of BAL against molds, such as *Colletotrichum* spp., suggesting a competitive role in the microbial stabilization of the beverage [156].

Technological conditions, such as container type (e.g., clay, wood, or steel), temperature, and the use of traditional inoculants, directly influence the composition and persistence of yeasts and fungi. Fermentation in porous containers, such as clay vats, can favor the presence of adapted native species. Open environments increase the diversity of wild yeasts [168,169]. Additionally, acidification produced by LAB regulates the dominance of acid-tolerant yeasts, such as *Candida*, *Hanseniaspora*, and *Pichia*. These yeasts coexist in equilibrium with bacteria and fungi in complex fermentation matrices. From a functional perspective, some *Saccharomyces* and *Pichia* strains have demonstrated probiotic potential due to their ability to withstand simulated gastrointestinal conditions and modulate intestinal microbiota [166]. They have also been shown to participate in reducing antinutritional compounds, such as phytates, and in the production of bioactive peptides, thus increasing the bioavailability of nutrients.

#### 5.1.3. Pathogenic Microorganisms

The spontaneous fermentation of traditional corn-based beverages such as pozol, chicha, atole agrio, and sendecho involves a complex interaction of beneficial and pathogenic microorganisms. While these beverages have traditionally been consumed without serious health consequences, studies have found pathogenic bacteria, including *Escherichia coli*, *Staphylococcus aureus*, *Salmonella* spp., and *Listeria monocytogenes*, in samples collected under unsanitary conditions [149,156]. The lack of standardization in artisanal fermentation processes, the use of contaminated utensils, and the use of untreated water increase the risk of contamination, particularly in the initial stages, before beneficial microorganisms reach sufficient concentrations to control pathogens.

Using contaminated water sources is an important factor in introducing undesirable microorganisms. For instance, Gagnon et al. [169] demonstrated that the microbiological quality of water used to produce chicha in Ecuadorian indigenous communities directly affects the diversity of microorganisms present. They found evidence of coliform bacteria in chicha made with river water. Similarly, Sainz et al. [171] isolated 73 *E. coli* strains from pozol samples at various fermentation stages, some of which belonged to the pathogenic ETEC, EPEC, and UPEC groups. Many of these strains exhibited typical adhesion patterns in HEp-2 cells and possessed virulent genes and antibiotic resistance, highlighting the importance of implementing strict hygienic practices when preparing these beverages.

However, controlled fermentation provides a natural defense against the proliferation of pathogens. LAB, including *Lactiplantibacillus plantarum*, *Leuconostoc mesenteroides*, and *Weissella confusa*, acidify the medium and produce bacteriocins that inhibit pathogens, such as *Salmonella Typhimurium* and *L. monocytogenes* [143,156]. If process conditions are not optimal, however, with regard to temperature, acidity, or fermentation time, these barriers may be insufficient, allowing pathogenic microorganisms to persist for critical periods.

Microbiological studies have demonstrated the survival of certain pathogens in these beverages. For instance, *Salmonella Typhimurium* has demonstrated greater tolerance to the acidic environment in chicha than *Shigella* spp., remaining viable for several hours [153]. *Listeria monocytogenes* can survive for several days in beverages stored at 4 °C without actively multiplying, yet still pose a latent risk to vulnerable populations [163]. These observations suggest that a low pH alone does not ensure the complete elimination of pathogens and that other factors, such as the antimicrobial compounds produced during fermentation and storage, also play a significant role.

To ensure the microbiological safety of these beverages while maintaining their traditional character, it is recommended that good manufacturing practices (GMP) and good hygiene practices (GHP) be adopted and that the use of indigenous starter cultures with antimicrobial properties be considered [117,172,173]. Critical steps in minimizing risks include cleaning and disinfecting utensils, using potable water, and carefully selecting raw materials. Similarly, conducting microbiological challenge studies can validate the safety of the fermentation process by confirming that the system’s intrinsic and extrinsic parameters are sufficient to inhibit or eliminate pathogenic microorganisms [145,157]. These strategies enable the articulation of food safety with the cultural and functional valorization of fermented corn beverages.

## 6. Nutrients Biotransformation and Production of Value-Added Compounds

**Figure 2:**
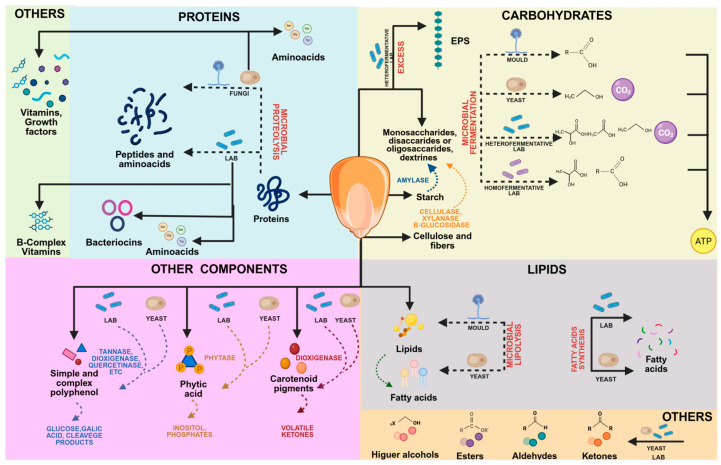
Overview of the main molecules in microbial biotransformation during maize fermentation.

During corn fermentation, simultaneous and complex activities develop. Molecule biotransformation always depends on raw material characteristics (maize variety, season, cultivars, etc.), pretreatments, employed inoculum or consortium interactions, and the type of final product. During traditional or non-controlled fermentations, plenty of microbial genera co-exist; nevertheless, the microbiome evolves with a few major genus prevalences. The main molecule biotransformation in corn-fermented products is shown in Figure 2.

### 6.1. Impact on Carbohydrate

Firstly, the carbohydrate content in raw corn is mainly starch, with relatively low to moderate quantities of free sugars [92,174]. A few pre-steps could help change the corn sugar content before the fermentation process, including nixtamalization, germination, or amylase treatment [8]. López-Sánchez et al. (2023) observed that during the initial stage of the pozol fermentative process, a great variety of microbial genera co-exist, but after the first nine hours of fermentation, the *Streptococcus* genus predominates [165]. During the corn fermentative procedure, amylolytic activities in microbial sources result in an important issue [8]. Less complex sugars, including monosaccharides, disaccharides, and oligosaccharides, are consumed by numerous microbial genera; nixtamalization, some soaking procedures, and initial microbial charge may promote a reduction in free sugars, which makes starch the main carbon source. The *Streptococcus* genus presents relevant amylolytic activity, which could explain its predominance in pozol fermentation [165]. Mao et al. (2024) used *L. plantarum* to generate corn-fermented flour [175]. After 3 days of fermentation at 45 °C, the amylose percentage decreased. The authors related this behavior to the enzymes and organic acids produced by *L. plantarum*, which promote hydrolysis in the starch amorphous region [175]. Metagenomic analysis shows abundant genes related to the production of carbohydrate-degradation enzymes in pozol fermentation, especially the α-amylases clan, but also plant wall, sucrose, and fructans degradation enzymes [165].

Masehlele et al. (2023) developed a corn fermented drink using kefir grains or yogurt starter cultures (*L. plantarum* and *Streptococcus thermophilus*) [176]. After the fermentative procedure, they observed elevated quantities of another class of sugars besides glucose, including arabinose, rhamnose, and mannose, especially in fermented beverages compared with unfermented corn-based drinks [176]. This class of carbohydrates may result from the degradation of corn fiber, including cellulose and hemicellulose. Other authors report a strong reduction in the arabinoxylan fraction during corn-bran spontaneous fermentations [177]. Despite LAB representing the most abundant microbial group, there exist differences in how they colonize the substrate. LAB possess different carbohydrate metabolisms. Homofermentative LAB convert glucose into lactic acid as the almost sole product (~80%) through the Embden–Meyerhoff glycolysis route, while heterofermentative LAB convert glucose to lactic acid by the phosphoketolase pathway, resulting in lactic acid, ethanol, and acetic acid mixtures [178]. The resulting organic acids promote a decrease in pH environment during fermentation. Heterofermentative strains normally promote a slower pH decrease compared to homofermentative strains due to the elevated lactic acid production. After 30 h of pozol fermentation, the pH drop is sharper. Some authors attribute this behavior to a shift from heterofermentative LAB at the earlier fermentation stage to homofermentative LAB in the later stages [165]. Heterofermentative LAB are common in plant-derived fermented foods; this group presents the ability to catabolize pentose (e.g., xylose and ribose) using the same hexose metabolic pathway. Despite glucose–lactate production being considered an energy-poor pathway, this could be improved by phosphorolytic cleavage of disaccharides or by using acetyl acetate as electron acceptors, whereas homofermentative organisms degrade maltose or fructose only after glucose depletion [178,179,180]. Furthermore, LAB in a medium rich in monosaccharides could synthesize EPSs [8]. EPSs are high-molecular-weight hetero- or homo-polysaccharides with a slimy texture, slightly attached to external microbial membranes, but can be completely secreted to the external environment. EPSs provide protection, adhesion, and biofilm capacity activities and contribute to changing physical, organoleptical, and functional properties in fermented foods and beverages [181]. EPSs raise the viscosity in cereal-based fermented beverages [182]. The studies from López-Sánchez et al. (2023) detected the presence of activated genes that produce EPSs between 9 h and 48 h of corn dough fermentation in pozol [165]. Grosu-Tudor et al. (2019) analyzed the content of EPSs in “borş”, a wheat–white bean–corn kernel fermented drink using different homemade and commercial products as starter cultures [183]. They detected up to 1.2 EPS g/L, also observing that the EPS content is higher at 12 h [183]. Masehlele et al. (2023) observe higher viscosity in corn-based fermented drinks than in the control [176]. The recovered EPSs possessed glucose, xylose, and rhamnose [176]. Multiple studies show that cereal-based products are a source of EPS-producing microorganisms [184,185].

Moreover, yeast also contributes to transforming carbohydrates during maize fermentation. Yeast ferments hexose into ethanol and carbon dioxide [186], a key feature especially in alcoholic beverages. But it also acts synergistically with LAB [8,106,141,187]. Yeast normally ferments a wide variety of carbohydrates broadly found in ripening fruits and cereals, including sucrose, glucose, fructose, maltose, and maltotriose, depending on yeast species [186]. Also, the acidic environment generated by LAB damages starch granules, helping yeast to hydrolyze starch [8,106,141,187,188]. Sucrose, maltose, maltotriose, and amylose degradation contributes to monosaccharide accumulation as glucose and fructose. Posteriorly, homo- and heterofermentative LAB could use glucose, and fructose could be reduced to mannitol by heterofermentative LAB [179,189]. Despite the carbohydrate–metabolic conjugation, the yeast count is lower than LAB in this class of fermented products, especially in sourdough-like products, where commonly present a 100:1 LAB–yeast proportion [190]. Some studies show that LAB–yeast co-culture generates benefits for LAB growth [191]. The yeast provides LAB, important molecules such as pyruvate, amino acids, and vitamins [8,106,141,187]. Regarding corn-fermented alcoholic products, a mixture of yeast–LAB predominates. The species *S. cerevisiae* is highly predominant in this class of beverages, but some non-*Saccharomyces* are present. Non-saccharomyces species produce a low rate of alcohol compared with *S. cerevisiae* [192]. In addition, the LAB interaction diminishes ethanol production by yeast due to carbohydrate competition and an acidic environment, which could explain the low alcoholic grade and complex flavor in corn-based products.

Food-fermented products are recognized as an ancient and relevant group of foods. The actual demand and their intention to be industrialized confront the bottleneck of complexity between microbial consortia and nutrient–product dynamics. Modernity allows us to disentangle the multidimensionality of fermentative procedures from the gene, enzyme, and metabolite perspectives [193] and how interactions such as mutualism, commensalism, competition, and amensalism maintain the richness of these food products [187]. The novel omics approach empowers food technology expression in the form of novel nutritious and highly bioactive products, industrialized processing, and designed, homogenized cultures.

### 6.2. Impact on Protein

During the corn fermentative process, different protein patterns have been observed in the final products.

Anaemene & Fadupin (2020) compared the nutritional composition of different quality protein corn flours [194]. The 72 h naturally fermented corn possessed a slightly higher crude protein content, 10.44%, compared with 10.04% from raw corn. During these studies, different germination and fermentation processes were contrasted. Germination for 72 h did not affect protein content, but when the fermentation procedure was developed after germination, the crude protein content was lower. Further, a 24 h extended fermentation process after germination decreased protein to 9.92% while a 48 h fermentation extension resulted in 9.12% [194]. Also, Decimo et al. (2017) employed two commercial corn brans (C1 and C2) to create sourdough [177]. After fermentation, C1 did not show statistically different quantities in crude protein, while C2 showed a ~1.3% decrease. In both cases, the microbial evolution mode was distinctive, with marked microbial shifts in early and mature sourdoughs [177]. On the other hand, Ogodo et al. (2017) reported increased protein quantities in fermented corn flours (12.2%) using LAB consortia compared with an unfermented product (9.2%) [195]. Pre-treatments could elevate starch and other polymers’ hydrolysis, which promotes corn-zein exposure for proteolysis and microbial metabolism, during fermentative procedures. Also, the class of microorganisms involved, or their succession pattern, could strengthen or reduce the protein quantity due to their proteolytic activity arrangement or biomass production capacity.

During corn beverage or food fermentation, the protein content can vary depending on several factors, including the class of microbial communities, corn pretreatments, endogenous corn protein quality, or even fermentation time; however, the fermentation process enhances protein quality [196].

One of the main mechanisms involved in protein biotransformation includes hydrolysis by microbial proteolytic enzymes. LAB employ corn proteins to satisfy their nitrogen requirements using a well-designed proteolytic system formed by cell-bound proteinases, although these can also be extracellular, peptide transporters, and intracellular peptidases, which, in general, allow the growth of this species in protein-based media [197].

Castillo-Morales et al. (2005) observed an increase of 31.9 g/kg in soluble protein in “Chorote”, a cacao–corn sourdough, after 9 days of fermentation, compared to 6.1 g/kg at 0 h [198]. The LAB system turns protein substrates into peptides and free amino acids to achieve activities such as stress resistance, energy generation, intracellular pH maintenance, and protein synthesis [197]. Zein solubilization in fermented beverages is not exclusive to LAB. Hayta et al. (2001) prepared Boza, a traditional African fermented drink, using a mixture of equal parts of corn and wheat flour, diluted in water, and fermented using pressed bread yeast [199]. They observed an enhancement in protein solubility from 0.72 mg/L to 4.60 mg/L after 30 h of fermentation [199]. Proteolysis in corn protein generates peptides that can present diverse bioactive properties, including antioxidant, anticancerogenic, antihypertensive, antimicrobial, and antidiabetic, among others [37]. The protein fragment’s functionality depends on its amino acid composition, sequence, and chain length, which is related to the protease cleavage site and protein source. Normally, a combination of diverse enzymes can help to improve peptide functionality [200]. In the case of corn-fermented products, high microbial biodiversity can help to generate a broad spectrum of cleavage sites in proteins.

Rebaza-Cárdenas et al. (2023) analyzed the chemical composition of six different Peruvian fermented beverages called “Chicha de siete semillas”, which include different classes of cereals and legumes as fermentative substrates, and the free amino acids (AAs) were quantified [145]. Two beverages demonstrated nearly 60 mg/L of total AAs. However, elevated quantities of glutamic acid and proline were quantified in all samples [145]. Glutamic acid (21–26%) and proline (10%) were abundant in zeins, the primary proteins in corn [65]. This could be related to the hydrolysis of this class of storage proteins by microbial growth. Also, fourteen of the twenty essential AAs were present in these beverages, with lysine and tryptophan detected in two samples, strongly associated with genera such as *Lactobacillus* sp., *Streptococcus* sp., *Weissella* sp., *Leuconostoc* sp., and *Zymomonas* sp. [145]. Besides protein hydrolysis, the correct selection of starter culture could add to food-essential AAs. Olakunle et al. (2023) realized large microbial screening among LAB and yeast from ‘ogi’ paste processing and selected *L. brevis* X043 and *Saccharomyces cerevisiae* OY4 as starter cultures [201]. The unfermented dehulled corn possessed lower quantities of lysine and methionine than the fermented one using the selected cultures, with a great improvement by 2% of glucose addition. Cui et al. (2012) increased the lysine content of the “Amahewu” drink by fermenting four corn varieties, including waxy bicolor and extra-sweet yellow corn, inoculated with a yeast [202]. All varieties increased their lysine content after fermentation [202].

Rizo et al. (2021) realized a metaproteomic analysis in pozol samples and identified that nearly 20.16% of bacterial origin proteins are associated with AA metabolism, which includes proteases (aminopeptidases and carboxypeptidases) for AA recycling; enzymes related to arginine biosynthesis from ornithine, oxaloacetate metabolism for aspartate, or asparagine production and the precursors of lysine and other AAs have also been identified [203].

Protein enhancement in fermented cereal products could be attributed, furthermore, to biomass production (yeast, bacteria, or mycelia), microbial protein (enzymes), and AA synthesis. Decreases could be attributed to the degradation of support for the complex microbiome in this class of fermentative procedure [204].

### 6.3. Impact on Lipids

A great number of studies on fermented cereal-based products refer to a reduction in lipid content from the fermented foods and beverages compared to the raw cereals [204]. Anaemene & Fadupin (2020) observed that during corn fermented flour production with 72 h fermentation and 72 h germination treatments, the crude fat content decreased at 4.30% and 3.20%, respectively, compared to the 4.72% from raw corn; nevertheless, the germination–fermentation successive steps in grains allow for lipid gain in flours, with increases from 3.40 to 3.80% with 24 and 48 h of fermentation time [194]. Normally, during corn germination, endogenous enzymes allow the hydrolysis of different storage molecules, including carbohydrates, proteins, and lipids. The corn lipids are stored as triglycerides, and as germination is realized, these compounds are hydrolyzed by lipases and transformed into organic alcohols and fatty acids to finally obtain energy for multiple metabolic activities [205]. This could explain the decline in crude fat after corn germination. At the same time, microbial growth is also involved in the lower quantities of lipids in untreated corn grains by a similar mechanism: energy uptake by lipid metabolism.

During metaproteomic analysis of pozol fermentation, the total protein associated with lipid metabolism was the ninth most abundant group of bacterial proteins, while among fungal proteins, it took seventh place [203]. Terefe et al. (2021) fermented corn flour over 48 h using different classes of inoculum *L. plantarum* (LP), *S. cerevisiae* (SC), both strains together (LP + SC), and, as a control, non-inoculated or natural fermentation (N) [206]. They observed that in all cases, fat content decreased, but during LP + SC, the decrease was more prominent [206]. During Rizo et al.’s (2021) studies, the enzymes involved in fatty acid degradation into Acetyl-CoA were identified [203]. Acetyl-CoA represents a key molecule in the tricarboxylic acid (TCA) cycle, involved in carbohydrate synthesis, which could be critical in conditions such as low simple sugar concentrations [203].

Castillo-Morales et al.’s (2005) study showed a 26 g/kg of crude fat gain after 9 days of fermentation for corn–cacao sourdough “chorote” production [198]. Also, microbial kinetics displayed a rise in yeast, mold, and amylolytic bacteria count [198]. Chelule et al. (2010) formulated an “amahewu”, a South African corn-based porridge [207]. The formulations consisted of flour, yeast, and sucrose variants, with salt and corn as core ingredients. The formulas with yeast and flour addition showed increased protein quantities and a favorable protein profile, which is linked to increased yeast biomass further enhancing lipidic content. The fermenting microorganisms synthesized lipidic molecules that could be incorporated into their membranes, which could explain this behavior [207].

One product from carbohydrate catabolism is Acetyl-CoA, which is also a precursor for fatty acid synthesis. The first step in this metabolic route is the carboxylation of acetyl-CoA, carried out by acetyl-CoA carboxylase, an enzyme identified in the metaproteomic analysis of pozol. Additionally, different fatty acid synthases from both prokaryotic and eukaryotic origins have been identified. These enzyme classes are fundamental for cellular membrane lipid synthesis [203].

### 6.4. Impact on Phytochemicals

The polyphenolic fraction in corn is distributed throughout the entire kernel, presenting a free and a bound fraction, and during corn fermentation, this polyphenolic content changes. Decimo et al. (2017) observed an approximately two to five times increase in ferulic acid content within two different native corn brans during a 12-day sourdough-like fermentation [177]. Also, Zhang et al.’s (2025) study showed how *Bacillus subtilis* submerged fermentation in corn-based media generated an important accumulation of free phenolic content (FPC) with a decrease in bounded phenolic content (BPC) [208]. During fermentation, enzymes such as amylase, protease, cellulase, xylanase, and β-glucosidase were quantified. They observed that amylase and protease activities presented elevated quantities during the first 48 h, with a subsequent decrease, while the cellulase peak was delayed to 96 h with a posterior decline. Xylanase and β-glucosidases were present at the lowest values, but with an increasing tendency to 168 h. In cereals, a great proportion of the total phenolic content is the bound type (50–86%) [208], normally attached by covalent bonds forming ether or ester linkages to cell components, including lignin, cellulose, proteins, hemicellulose, or pectin in fruits, vegetables, and cereals [209]. Therefore, the hydrolysis of a cell-wall component induces bound phenolic release [202]. The necessary enzymes to develop corn matrix degradation have been reported in several LAB and yeast strains involved in fermented food and beverage production [8].

Among cereals, the major phenolic groups are phenolic acids (hydroxybenzoic and hydroxycinnamic acids), flavonoids, and tannins. Tannins are elevated-molecular-weight molecules that contribute to cereal pericarp color and are classified as hydrolysable (ellagic acid, gallic acid units) or condensed (flavonoid units) [210]. During cereal fermentation, tannins are hydrolyzed into small units, displaying a reduction in their quantification. Anaemene & Fadupin (2020) observed a depletion in tannin content after consecutive 72 h germination and 48 h fermentation in corn kernel [194], while Terefe et al. (2021) observed a reduction of 75% in tannins employing a yeast–LAB co-culture in corn-flour fermentation, presenting the highest value compared with monocultures and natural fermentation [206]. Tannins are considered anti-nutrient compounds due to their ability to form complexes with molecules such as proteins, cellulose, starch, and minerals, among others, limiting their digestibility. Their mass is directly related to their biological effects, where small compounds present fewer antinutritional effects [211,212]. Tannin hydrolysis is not fully studied in fermented corn beverages and foods. Multiple microbial species found in this class of products have been reported as tannin-degrading species, including *L. plantarum* [212], *S. cerevisae*, *Pediococcus* sp., *Weissella* sp., and *Lactobacillus brevis*, among others [211]. The enzymes involved in Gallo tannin degradation include tannin-acyl hydrolase, which hydrolyzes galloyl ester bonds, resulting in gallic acid and glucose [212] while condensed tannins involve dioxygenases [213].

Besides the release of polyphenolic components in fermentation media, they can also be biodegraded by microorganisms. Liang et al. (2023) analyzed fermentative products for different common polyphenols using *Aspergillus oryzae*, *S. cerevisiae*, and *L. plantarum* to elucidate their derivatives [214]. During the study, they observed that *L. plantarum* transforms polydatin to resveratrol and resveratrol 4-methyl ether, cyanidin-3 glucoside to ferulic acid, ferulic acid to cinnamic acid, and Rutin to protocatechuic acid. *S. cerevisiae* transforms ferulic acid into vanillin, and both strains hydrolyze tannic acid. The author proposed a wide range of enzymes involved, including decarboxylases, β-glucosidases, α-rhamnosidase, quercetinase, tannase, and esterase [214]. Mikulajová et al. (2024) generated two fermented corn-mashes, using *Lactococcus lactis* ssp. lactis, *Lactococcus lactis* ssp. cremoris, and *Streptococcus thermophilus* combined with a different *Bifidobacterium* sp. Strain [215]. They observed that the phenolic compounds’ profiles changed with storage. They also observed that quantification in the phenolics was strain- and media-component-dependent and showed a general decrease in protocatechuic, p-coumaric, vanillic, ferulic, and caffeic acids [215]. This class of phenolics can be degraded by decarboxylases and reductases.

Other important components in corn are pigments such as carotenoids, present especially in red or yellow varieties. Song et al. (2021) analyzed the effect of different corn treatments on multiple carotenoid content and bioaccessibility after simulated digestion [216]. They observed that carotenoid retention was higher in boiled corn (79.0%), followed by fermented porridge (73.8%) and fermented corn (68.4%). Treatments enhanced the extractability of carotenes after digestion compared with raw samples. Carotenoids are typically bound to biomolecules, such as proteins in cell walls. All the treatments could promote a softening or breakage of these components, thereby enhancing absorption after digestion [216]. Uenojo & Pastore (2010) reported a microbial transformation of β-carotene, leading to the generation of volatile ketones with fruity–floral–fermented–sweet aromas such as β-ionone (1), β-damascone (2), β-damascenone (3), pseudoionone (4), and 1,1,6-trimethyl-1,2,3,4-tetrahydronaphthalen (TTN) (5), where (1) was the most abundant [217]. Bento-Silva et al. (2021) reported the presence of α-ionone in corn flour and broa bread [218]. Carotenoid degradation could be driven by dioxygenase, among other mechanisms. If this is the case, the relation between fermentation and this class of volatile compounds, which is not fully studied, could be implicated in the development of some aromas in corn-based fermented products [218].

### 6.5. Microbial Metabolite Production During Fermentation

Corn is a natural source of water-soluble B-complex vitamins (thiamin, folic acid, riboflavin, pyridoxine, and pantothenic acid), present mainly in the aleurone layer but in low quantities [8,219]. Nevertheless, it lacks vitamin A and cobalamin [207]. Some traditional cereal processing activities even promote a major decrease in these molecules; nevertheless, fermentation boosts their concentration.

Obatolu et al. (2016), after formulating a fermented corn drink (sekete), conducted a vitamin content analysis after four weeks in cold storage [220]. They observed, in general terms, an increase in niacin content compared with raw yellow corn, from 0.37 mg to 0.50 mg and 0.53 mg per 100 g of product in unpasteurized sweetened non-alcoholic samples and unpasteurized plain alcoholic samples [220]. Also, Ongol et al. (2013) observed a niacin content impact after fermentation in two corn flours [196]. The germination process before fermentation promoted a 10-fold increase in both corn varieties. Chawafambira et al. (2021) compared mutwiwa (corn-fermented porridge) formulated with white corn and a biofortified corn variety ZS242 (provitamin A) with high carotenoid content [221]. After spontaneous fermentation, the fortified corn-porridge showed 9 µg retinol activity equivalent/100 g and 0.54 mg vitamin C/100 g, while the white corn-based porridge did not show vitamin A and had lower vitamin C [221]. Although the studies do not fully relate the fermentative procedure to vitamin A presence, the use of fortified or high carotene content corn varieties could influence the cleavage of these compounds to generate vitamin A. Some studies report that this cleavage could be realized by gut microbiota employing β-carotene-15,15′-oxygenase [222].

It has been reported that LAB produce vitamin B complexes, including cobalamin, folate, riboflavin, and thiamine. Yeast also supports these kinds of biologically active molecules in the fermenting media; nevertheless, occasionally, a decrease could also be present after being consumed by other microbial species [8,219], considering the complex microbial balance in fermented foods

Fermented foods and beverages normally present lower undesirable bacterial counts. Castillo-Morales et al. (2005), considering chorote microbial kinetics, observed how the coliform bacterial number decreased until it disappeared on the sixth fermentation day [198]. The further selection of bacterial communities in fermented food is shaped by several factors, including the low pH generated by organic acids produced by LAB and the presence of antimicrobial compounds such as hydrogen peroxide or bacteriocins [8]. Bacteriocins are ribosomal-produced peptides that exhibit antimicrobial properties and are excreted into the microbial growing media. LAB with bacteriocin production potential are widely reported, including *L. plantarum*, *L. lactis*, *Lactococcus sake*, *L. paracasei*, and *Pediococcus acidilactici*, presenting antimicrobial effects against food-borne pathogens such as *Escherichia coli*, *Salmonella enterica*, and *Pseudomona aureginosa*, among others. Bacteriocin production can vary in LAB, showing different antimicrobial spectra; also, one strain can produce multiple classes of bacteriocins [197]. Ogundare et al. (2021) isolated LAB from fermented corn slurry [223]. The crude extract from liquid fermentation showed an antimicrobial effect against several pathogens, including *E. coli*, *S. aureus*, and *B. subtilis*. The addition of this crude extract to orange juice diminished the microbial count. The extract was stable at different pH levels and was inactivated by trypsin [223]. Rasheed et al. (2021) used an *L. plantarum* (BZ532) bacteriocin-producer strain isolated from a Chinese cereal fermented drink and incorporated it into a home-made millet fermented drink “Bozai”, proving that the incorporation of the bacteriocin-producing strain decreased CFU/g of *S. aureus* and *E. coli* compared to the non-bacteriogenic *L. plantarum* strain [224]. Also, the Boza pH was elevated to 6.0, and the disc diffusion test showed that Boza inhibited *S. aureus* and *E. coli* with no effect in *Salmonella* sp., [224]. Diverse authors have isolated bacteriocin–LAB producers from corn fermented products, including ogi, poto poto, sha’a, and doklu [8,225].

### 6.6. Antinutritional Molecules Reduction

Cereals, including corn, have been reported as a rich source of nutrients; nevertheless, a group of molecules can lower their bioavailability. Phytic acid (myo-inositol 1,2,3,4,5,6-hexakis dihydrogen phosphate or its de-hydrogenated form (phytate)) in plants is recognized as a major source of phosphorus; nevertheless, it promotes non-soluble complexes with minerals at intestinal (light-alkaline) pH, but also can bind to lipids and proteins [8,226]. Phytic acid hydrolysis generates molecules with lower binding capacities [8]. Terefe et al. (2021) showed a phytate reduction from 300 mg/100 g to 100 mg/100 g during the fermentation of corn flour enhanced by LAB–yeast co-culture [206]. Also, Sun et al. (2024) fermented a by-product derived from wet milling for starch production, mainly composed of germ and bran with a *Bacillus subtilis* strain, and observed that during the first 48 h at pH 5–6, the phytate reduction was pronounced [227]. The authors reported 8.2 ± 0.24 U/g of phytase activity in the fermented product [227]. Multiple reports showing a reduction in phytase activities have been developed for corn fermented products. The main mechanism involves extracellular phytase production by microorganisms and the activation of endogenous corn phytase by the pH drop during fermentation, allowing the bioavailability of multiple minerals in fermented foods [8,177].

Protease inhibitors are another class of antinutrient compounds, present in cereals and other plant-derived foods, that are normally small and water-soluble proteins that affect protein hydrolysis during digestion, lowering the real nutritional value of plants as foods [228]. There are reports linking protease inhibitor reductions with plant-based fermented foods. Adeyemo & Onilude (2013) used *L. plantarum* strains isolated from fermented cereals to reduce antinutrients in soybeans, significantly decreasing tannins, phytates, and trypsin inhibitors compared with the raw matrix [229]. Terefe et al. (2021) also observed a reduction in trypsin inhibitors from 55 mg/100 g to nearly 20 mg/100 g in fermented corn flour after 48 h [206]. Another class of methods could enhance the reduction in this class of antinutritional molecules; nevertheless, deeper studies need to be developed.

## 7. Final Remarks

The chemical composition and nutritional value of corn varieties that diverge in pigmentation are different. This composition is affected by genetic, environmental, cultivation, and transformation process factors. Consequently, the physicochemical, nutritional, and microbiological characteristics of the final product are altered. Corn fermentation for beverage production induces a set of physicochemical transformations, unlocking great nutritional potential in one of the most consumed staple cereals worldwide. The microbial organisms present in traditional fermented products possess a biochemical machinery which can contribute to alleviating or minimizing the consequences of poor modern diets. Moreover, the extended understanding of complex microbial interactions and matrix transformation in food opens up new possibilities for developing controlled fermentative technologies which allow us to retain traditional beverages’ benefits while generating nourishing, safe, and innovative foods.

On the other hand, despite growing interest in fermented beverages made from pigmented corn, key gaps in research remain. Currently, there is limited knowledge about how various pigments, mainly anthocyanins and carotenoids, interact with fermenting microorganisms and modify the sensory profile, color stability, and functionality of the final product. There is also a lack of information on the relationship between the nutritional profile and pigmentation of the grain. Furthermore, the absence of standardized methodologies for quantifying pigments before, during, and after fermentation, as well as studies on their bioavailability and behavior during processing and storage, is noteworthy. Similarly, there is considerable variability in the methods used to produce various corn-based fermented beverages, which complicates their analysis. In the future, advances are expected in the development of varieties with higher bioactive compound content, the application of omics technologies to better understand pigment transformations, and the design of starter cultures that preserve these compounds.

## Figures and Tables

**Table 2:** Fatty acids profile of different corn varieties.

Corn Variety (*Zea mays*)	Fatty acids (mg/g de TFA)						References
	PAA	STA	OLA	LNA	LLA	AAA	
White	11.00–15.00	2.00–4.00	33.00–44.00	0.50–5.00	34.00–52.00	0.35–0.55	[82,83]
Yellow	11.00–13.00	2.00–4.00	34.00–43.5	0.55–4.50	37.00–52.00	0.35–0.45	
Blue	2.86	4.30	52.21	n.d	44.85	n.d	[84]
Red	12.35–13.05	2.00–3.45	33.5–41.00	0.75–0.84	42.50–49.70	n.d	[73,85]

TFA, total fatty acids; PAA, palmitic acid; STA, stearic acid; OLA, oleic acid; LLA, linoleic acid; LNA, linolenic acid; AAA, araquidonic acid; n.d, not detected.

**Table 3:** Polyphenols, anthocyanins, and carotenoids profiles in different corn varieties.

Corn Variety (*Zea mays*)	TPC (mg GAE/100 g DW)	Polyphenols Profile	TAC (mg cy-3-glc equiv/100 g DW)	Anthocyanins Profile	TCC (µg β-carotene/100 g DW)	Carotenoids Profile	References
White	132.0–273.2	Ferulic acid, gallic acid, quercetin	n.d	n.d	n.d	n.d	[43,93]
Yellow	135.9	Gallic acid, caffeic acid, ferulic acid	n.d	n.d	675	Lutein, Zeaxanthin, Cryptoxanthin, α-carotene	[94,95]
Blue	85.6–294.1	*p*-coumaric acid, ferulic acids, chlorogenic acid	181.6–796.2	Cyanidin 3-glucoside Cyanidin 3-malonyl glucoside Pelargonidin 3-glucoside Peonidin 3-glucoside Pelargonidin 3-malonyl-glucoside	n.d	n.d	[96,97,98]
Purple	311.0–817.6	*p*-coumaric acid ferulic acid 8-5′-Ben DiFA	1.97–71.68	cyanidin-3-glucoside cyanidin-3-(6″- malonylglucoside) peonidin-3-(6″-malonylglucoside) pelargonidin-3-(dimalonylglucoside) peonidin-3-(dimalonylglucoside)	n.d	n.a	[99]
Red	141–217	Chlorogenic acid, gallic acid, dihydroxybenzoic acid, syringic acid	n.d	n.d	n.d	n.d	[100]

TPC, total polyphenols content; TAC, total anthocyanins content; TCC, total carotenoids content; GAE, gallic acid equivalents; cy-3-glc, cyanidin-3-glucoside equivalents, DW, dry weight; n.d., not detect; n.a, does not apply.

**Table 4:** Main microorganisms found in fermented corn-based beverages.

Fermented Beverages	Bacteria	Yeasts	Fungi	References
Pozol	*Lactobacillus plantarum*, *L. fermentum*, *L. delbrueckii*, *Lactococcus lactis*, *L. casei*, *Streptococcus suis*, *Clostridium* sp., *Acetobacter*, *Weissella confusa*, *Bacillus subtilis*	*Saccharomyces cerevisiae*, *Candida* spp., *Geotrichum candidum*	*Aspergillus*	[142,146,147,148,149]
Chicha de jora	*Lactobacillus*, *Leuconostoc*, *Acetobacter*	*Saccharomyces cerevisiae*, *Candida ethanolica*, *Pichia kudriavzevii*, *Hanseniaspora opuntiae*, *Wickerhamomyces anomalus*		[134,144,145,150,151,152]
Chicha ecuatoriana	*Lactiplantibacillus plantarum*, *Leuconostoc*, *Weissella*	*Candida intermedia*, *Candida parapsilosis*, *Pichia fermentans*, *Wickerhamomyces anomalus*		[144,152,153]
Champús	*Lactobacillus*, *Weissella*, *Leuconostoc*	*Issatchenkia orientalis*, *Saccharomyces cerevisiae*, *Pichia fermentans*, *P. kluyveri*, *Torulospora delbrueckii*, *Hanseniaspora* spp., *Zygosaccharomyces fermentati*	*Galactomyces geotrichum*	[154,155]
Tejuino	*Leuconostoc citreum*, *Weissella cibaria*, *Lactobacillus plantarum*, *L. fermentum*, *L. paracasei*, *L. pentosus*	*Saccharomyces*, *Candida*, *Pichia*, *Wickerhamomyces*		[124,129,156,157,158]
Atole agrio	*Weissella confusa*, *Lactobacillus fermentum*, *Lactobacillus plantarum*, *Lactococcus lactis*, *Pediococcus pentosaceus*			[117,159]

## Data Availability

No new data were created or analyzed in this study.
